# Co-infection of *Helicobacter pylori* with Epstein-Barr virus in gastric organoids enhances cell proliferation and morphogenesis

**DOI:** 10.1128/jvi.00928-25

**Published:** 2025-08-20

**Authors:** Lina Liu, Caixia Zhu, Shuxin Zhang, Yantao Duan, Yulin Zhang, Shujuan Du, Yuping Jia, Fang Wei, Daizhou Zhang, Dazhi Xu, Yuyan Wang, Qiliang Cai

**Affiliations:** 1 MOE&NHC&CAMS Key Laboratory of Medical Molecular Virology, Shanghai Institute of Infectious Disease and Biosecurity, Shanghai Frontiers Science Center of Pathogenic Microorganisms and Infection, School of Basic Medical Science, Qidong-Fudan Innovative Institute of Medical Science, Shanghai Medical College, Fudan University 58305, Shanghai, China; 2 Department of Gastric Surgery, Fudan University Shanghai Cancer Center 89667, Shanghai, China; 3 Shandong Academy of Pharmaceutical Sciences 636565, Jinan, China; 4 ShengYushou Center of Cell Biology and Immunology, School of Life Sciences and Biotechnology, Shanghai Jiao Tong University 200639, Shanghai, China; 5 Expert Workstation, Baoji Central Hospital, Baoji, China; University of Virginia, Charlottesville, Virginia, USA

**Keywords:** *Helicobacter pylori*, Epstein-Barr virus, gastric organoids, co-infection

## Abstract

*Helicobacter pylori *(*H. pylori*) is a key cause of chronic inflammation and gastric cancer, and Epstein-Barr virus (EBV) also contributes to gastric tumorigenesis. However, the role of EBV co-infection in *H. pylori*-related cancers remains unclear because of the lack of efficient *ex vivo* models. Organoids, which are derived from self-renewing stem cells, closely mimic *in situ* tissue structure and function, making them ideal for disease modeling. Here, we developed patient-derived normal gastric organoids (NGOs) and used high-accuracy microinjection to introduce EBV and *H. pylori* (NCTC11637 strain). We found that the co-infection of *H. pylori* and EBV induced a significant structural change and upregulated the expression of *TFF1*, *VIL1*, and *Lgr5* to promote cell proliferation and tissue morphogenesis. Analysis of transmission electron microscopy revealed an increase of *H. pylori* internal location in NGOs after co-infection. Moreover, all tested *H. pylori* strains isolated from patients displayed similar toxicities when co-infected with EBV. These findings provide a fundamental basis for evaluating pathogen toxicity, predicting disease progression, and advancing the clinical treatment of gastric cancer.

**IMPORTANCE:**

*Helicobacter pylori* (*H. pylori*) infection is a major contributor to chronic inflammation and the development of gastric cancer. Furthermore, Epstein-Barr virus (EBV) has been shown to play a role in the oncogenic process of gastric cancer by promoting chronic inflammation and increasing tissue damage. However, the mechanism by which co-infection contributes to gastric carcinogenesis remains unclear. In this study, we used patient-derived gastric organoids as a model to establish EBV-*H. pylori* co-infection using microinjection technology and found that co-infection causes significant structural changes and promotes cell proliferation. This model will not only contribute to a better understanding of the pathogenesis of gastric cancer but will also be important for drug efficacy evaluation and the development of new therapeutic approaches.

## INTRODUCTION

Gastric cancer (GC) is a significant global health issue, ranking fifth among all cancer types, with nearly 1.1 million new cases reported in 2020. GC is frequently diagnosed at an advanced stage and has a high mortality rate, making it the fourth leading cause of cancer-related deaths worldwide (1, 2). The etiology of GC is intricate and involves a broad range of risk factors, including demographic variables (e.g., sex, age, and family history) and environmental influences (e.g., high-salt diets and secondhand smoke) (3–5). Notably, *Helicobacter pylori* (*H. pylori*) infection is considered the most critical risk factor for GC (6). In 1994, the International Agency for Research on Cancer (IARC) designated *H. pylori* as a Class I carcinogen. In 2022, the United States Department of Health and Human Services confirmed it as a definitive carcinogen, underscoring the urgent need for preventive strategies and early detection methods to combat this devastating disease.

*H. pylori* is a gram-negative bacillus that causes chronic gastritis. Chronic inflammation can result in gastric mucosal atrophy and intestinal epithelial hyperplasia over time. These conditions can eventually evolve into severe gastroduodenal lesions, such as peptic ulcers, gastric mucosa-associated lymphoid tissue lymphomas, and GC (7, 8). The pathogenesis of *H. pylori* infection is attributed to colonization and virulence factors, along with their interactions with the host immune system and environmental elements. Through its helical structure and flagellar motility, *H. pylori* can penetrate the gastric mucus layer and colonize the gastric mucosa. However, the pathogenicity of *H. pylori* is not exclusively dependent on its flagella. Additional crucial virulence factors include cytotoxin-associated protein A (*CagA*) and vacuolar toxin A (*VacA*), both of which play a role in the pathogenicity of *H. pylori* (7, 9). *CagA*, a macromolecular terminal gene product synthesized in the bacterial cytoplasm, is directly injected into host cells via the Cag Type IV secretion system (*Cag* T4SS) (10). Upon delivery, *CagA* undergoes tyrosine phosphorylation at its Glu-Pro-Ile-Tyr-Ala (EPIYA) motif, which is typically located in its C-terminal region (11, 12). Irrespective of its phosphorylated state, *CagA* can activate various signaling pathways within host cells, influence cell growth and polarity, and stimulate epithelial cell proliferation and inflammatory responses, all of which are closely linked to the progression of GC (13, 14).

GC is a highly heterogeneous malignant tumor categorized into four subtypes: microsatellite instability (MSI), Epstein-Barr virus (EBV)-associated, chromosomal instability (CIN), and genomically stable (GS) (15). EBV-associated gastric cancer (EBVaGC) is distinguished by an EBV-CpG island methylation phenotype and constitutes approximately 10% of all GCs. EBVaGC is characterized by frequent mutations in PIK3CA, overexpression of PD-L1/2, and methylation of the CDKN2A/p16INK4A promoter while lacking hypermethylation of MLH1 (16). This highlights the significance of EBV as another key pathogen contributing to the development of GC.

EBV, or human herpesvirus type 4 (HHV-4), is carried by more than 95% of healthy adults worldwide and closely linked to the development of nasopharyngeal carcinoma and Burkitt’s lymphoma (17). The incidence of EBVaGC varies across regions, ranging from 1.3 to 30.9%, with a global clinical average of 10%. Recent studies have demonstrated that the mechanisms by which EBV infection contributes to gastric tumorigenesis primarily involve inflammatory responses in the gastric mucosal epithelium, inhibition of T-cell proliferation through high expression of PD-L1 via the IFN-γ signaling pathway, and hypermethylation of tumor suppressor genes (18, 19). Although both EBV and *H. pylori* have been confirmed as causative agents for gastric tumor formation, whether these two pathogens synergistically promote the development of GC remains under investigation largely due to the scarcity of effective *ex vivo* research models (20–22).

Organoids are three-dimensional (3D) cell culture systems that closely resemble the structure and function of the original tissue or organ *in vivo*. Depending on their cellular origin, organoids can be classified into three types: embryonic stem cell (ESC)-derived organoids, induced-pluripotent stem cell (iPSC)-derived organoids, and patient-derived organoids (PDO). PDOs, or patient-derived tissue-like mini-organs, represent a promising innovation for enhancing the efficiency of new drug development and improving tumor therapy outcomes. Composed of a three-dimensional cell mass cultivated from self-renewing stem cells in culture dishes, PDOs can mimic the *in situ* structure and function of tissues (23, 24), which have become increasingly utilized in disease modeling, high-throughput drug screening, and genetic manipulation (25–31). In this study, we established patient-derived tumor or normal gastric organoids (T/NGOs) *in vitro* and investigated the interaction between *H. pylori* co-infection with EBV and the host by using NGOs as a model and employing high-precision microinjection techniques. We found that the co-infection of *H. pylori* and EBV causes significant structural changes and promotes cell proliferation, which not only provides a better understanding of the pathogenesis of GC but also represents a potential system for evaluating drug efficacy and developing new therapeutic approaches.

## RESULTS

### *In vitro* constructed patient-derived NGOs and TGOs can highly simulate gastric tissue

**Fig 1:**
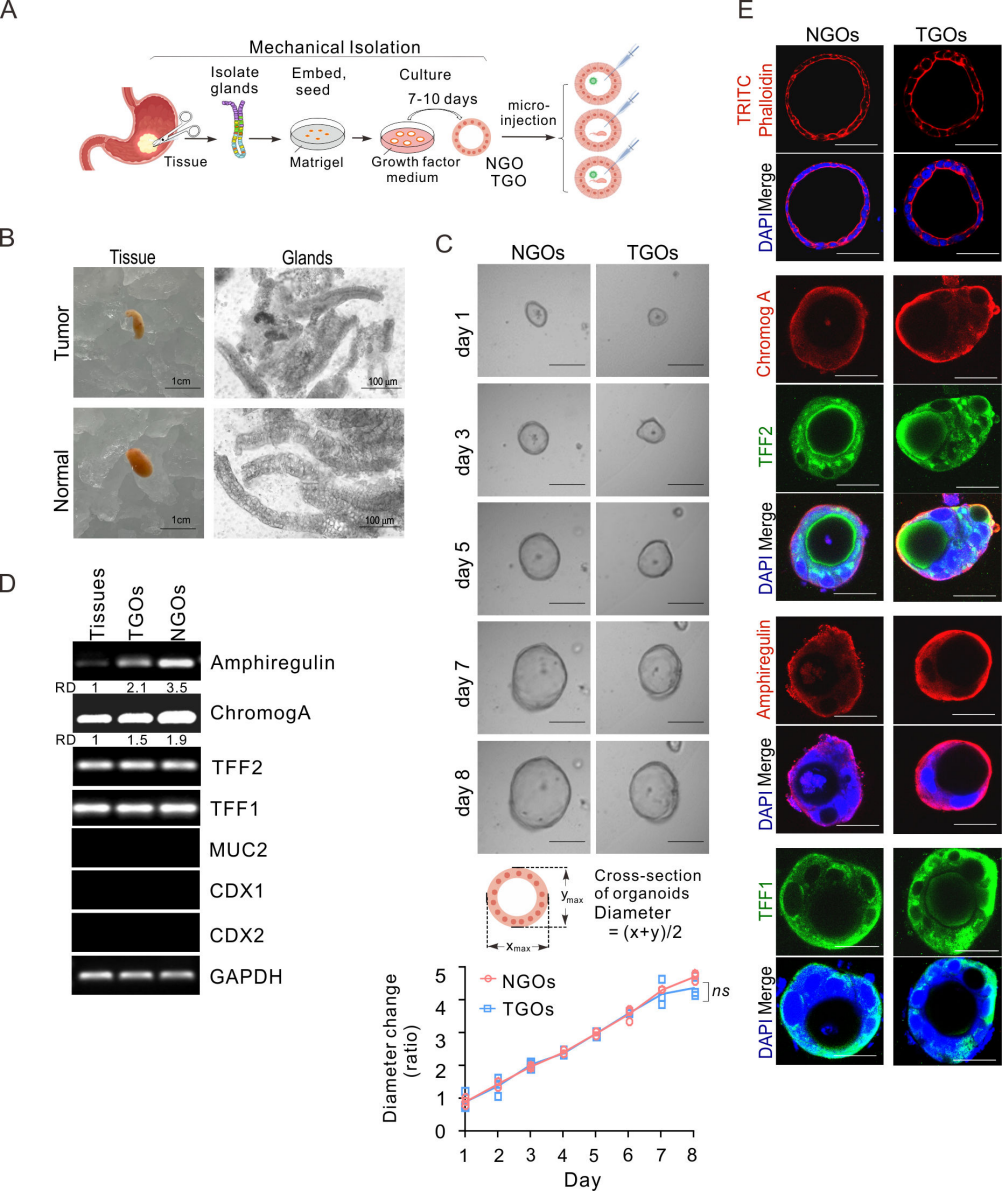
*In vitro* generation of patient-derived gastric organoids. (**A**) Workflow of human gastric organoid generation by mechanical isolation. The collection of gastric tumor and normal epithelium tissues, gland isolation, Matrigel matrix embedding, seeding, and growth factor supplement medium were included for culture of normal gastric organoid (NGO) and tumor gastric organoid (TGO), followed by micro-injection. (**B**) Representative morphology of gastric glands isolated from tumor and normal tissues. Scale bar, 1 cm (right panel) or 100 µm (left panel). (**C**) Representative morphology of primary TGO and NGO culture generation from panel B for 8 days post-seeding. Scale bar, 200 µm. Bottom panel: the growth curve of TGOs and NGOs (*n* = 3) evaluated by cross-section diameter in the first passage. *ns*, not significant. (**D**) Expression levels of different biomarkers in gastric adjacent tissues, TGOs, and NGOs. The total RNA extracts from TGOs and NGOs in panel C at day 3 were subjected to detection by reverse transcription PCR analysis for different biomarkers of parietal cells (Amphiregulin), endocrine cells (Chromog A), neck mucous cells (TFF2), surface mucous cells (TFF1), intestinal epithelial cells (MUC2), and intestinal specific nuclear transcription factors (CDX1, CDX2), along with GAPDH as an internal control. (**E**) Immunofluorescence analysis of TGOs and NGOs in panel C. DAPI is staining for the nuclei. TRITC phalloidin is staining for the membrane structure F-actin. Scale bar, 70 µm.

To determine the role of *H. pylori* co-infection with EBV in the development of GC, we used our previously reported mechanical separation method to extract gastric epithelial stem cells from both tumor and adjacent normal tissues from patients with GC (32). These stem cells were then cultured in a growth factor-rich medium to promote their differentiation into mature tumor or normal gastric organoids (T/NGOs) in Matrigel to maintain the three-dimensional structure of the cells (Fig. 1A). Upon mechanical stress, both tumor and normal gastric epithelial cells released elongated, U-shaped glandular structures (Fig. 1B).

To determine whether there were morphological differences between TGOs and NGOs, human primary gastric organoids were seeded and continuously cultured for 8 days to determine tissue morphology and size. The results showed that the vast majority of both gastric organoid structures, regardless of their origin, exhibited a three-dimensional morphology with a spherical shape and no significant difference in their growth patterns (Fig. 1C).

To determine whether both TGOs and NGOs can mimic the functions of gastric epithelial cells *in vitro*, we extracted mRNAs from NGOs and TGOs and performed PCR amplification to detect the expression profile of genes related to the cell types intrinsic to the gastric mucosa, including Amphiregulin (for parietal cells), Chromog A (for endocrine cells), TFF2 (for neck mucous cells), and TFF1 (for surface mucous cells), with intestinal markers, such as MUC2, CDX1, and CDX2, as internal controls. The results showed that the expressions of biological markers for parietal cells, surface mucus cells, neck mucus cells, and endocrine cells were similar in both NGOs and TGOs (Fig. 1D), albeit the slightly higher levels of Amphiregulin and ChromogA in NGOs than TGOs. In contrast, no expression of MUC2, CDX1, and CDX2 was observed. These findings indicate that both NGOs and TGOs effectively expressed biological markers specific to gastric mucus cells, and the gastric organoids we constructed were viable and did not undergo intestinal metaplasia. In the immunohistochemical analysis of nuclear and cytoplasm staining, including Amphiregulin, Chromog A, TFF2, and TFF1 biomarkers, we observed that the majority of human gastric organoid tissues had a spherical structure with a single layer of cells lining the interior and central lumen (Fig. 1E), which are capable of performing the functions of gastric glands.

### *H. pylori* strain NCTC11637 co-infection with EBV impairs gastric organoid morphology and growth

**Fig 2:**
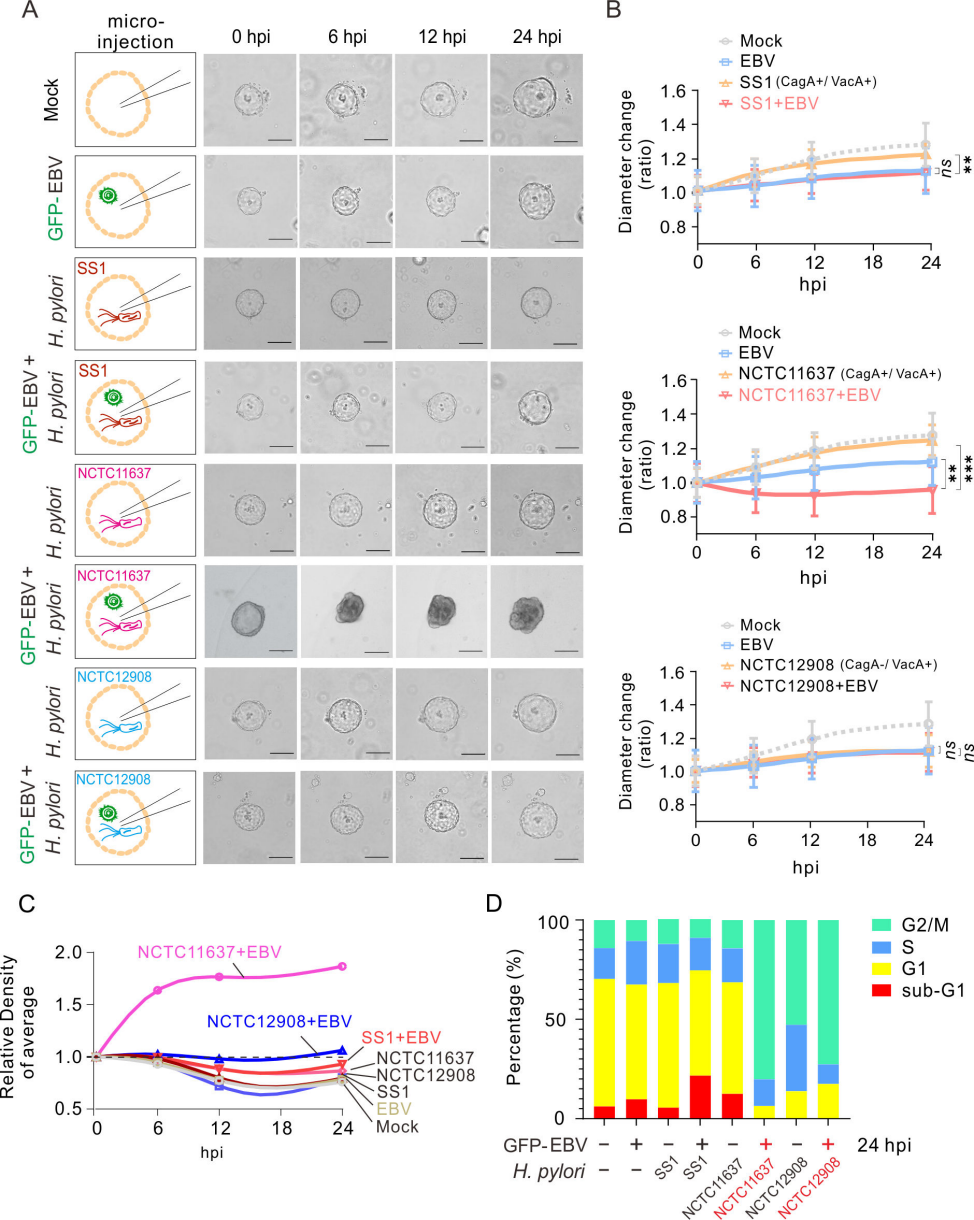
Effect of *H. pylori* co-infection with EBV via micro-injection on gastric organoid morphology and growth. (**A**) NGOs were individually micro-injected with GFP-tagged EBV virion particles (MOI = 10), different strains of *H. pylori* [SS1 (Cag A^＋^/Vac A^＋^), NCTC11637 (Cag A^＋^/Vac A^＋^), or NCTC12908 (Cag A^−^/Vac A^＋^)], or both, as indicated in the left panel, and the organoid morphology is visualized by microscopy analysis at 0, 6, 12, and 24 h post-infection (hpi). Scale bar, 100 µm. (**B**) The diameter ratio of NGOs micro-injected with *H. pylori* and GFP-EBV from panel A was evaluated by cross-section diameter analysis. (**C**) The relative density of NGOs micro-injected with GFP-EBV and *H. pylori* was evaluated based on the organoid morphology from panel A. (**D**) The cell cycle population percentage of NGOs micro-injected with GFP-EBV and *H. pylori* from panel A at 24 hpi was detected by flow cytometry analysis. The statistical analysis was done by ANOVA. **,* P *< 0.01, ***, *P *< 0.001, *ns*, not significant.

To investigate the effects of *H. pylori* (particularly the role of CagA as a key virulence factor) and EBV co-infection on GC, we used NGOs stably established *in vitro* as a “host” model with a high-accuracy microinjection technique to introduce different *H. pylori* strains of SS1, NCTC11637, and NCTC12908 (carrying with or without CagA) in the presence or absence of EBV virion particles into the lumen of organoids and visualize subsequent growth of the organoids for 24 h after infection. The results showed that both *H. pylori* strains SS1 (CagA+/VacA+), NCTC11637 (CagA+/VacA+), and NCTC12908 (CagA−/VacA+) and EBV infection alone or co-infection led to reduced growth of NGOs compared to the mock group (Fig. 2A and B). Notably, the *H. pylori* strain NCTC11637 (CagA+/VacA+), but not SS1 or NCTC12908, resulted in a significant loss of the normal spherical structure (displaying a loss of structural integrity, with crumpling, folding, and curling into a ball) of NGOs after co-infection with EBV at 6 h post-microinjection (Fig. 2A and B, middle panels). Nevertheless, despite this initial loss of structure, the organoids presented the capacity to grow and significantly increase in cell density along with the prolongation of infection time within 24 h compared to the EBV- or *H. pylori*-only groups (Fig. 2C).

To further address the effects of EBV and *H. pylori* co-infection on the growth of gastric organoids, we detected the cell cycle of NGOs infected with different strains of *H. pylori* (SS1, NCTC11637, and NCTC12908) in the presence or absence of EBV at 24 h post-microinjection. The results showed that co-infection of the *H. pylori* strain SS1 with EBV resulted in a prolonged sub-G1 phase in NGOs, whereas the co-infection of strain NCTC12908 with EBV led to a prolonged G2/M phase in NGOs (Fig. 2D). Intriguingly, the co-infection of strain NCTC11637 with EBV resulted in a significant increase in the G2/M population and a reduction in the sub-G1 and G1 populations compared to infection with the strain NCTC11637 alone (Fig. 2D, middle panels). This indicates that the impact of EBV and NCTC11637 co-infection on the growth of NGOs may be attributable to the stimulation of cell division and proliferation, which leads to a relative increase in organoid cell density. In contrast, the low proportion of subG1/G1 in NGO infection with NCTC12908 alone compared to that with SS1 and NCTC11637 could be attributable to the absence of the VacA virulence factor in NCTC12908.

### Morphology and cell density of NGO co-infected with EBV are related to *H. pylori* toxicity

**Fig 3:**
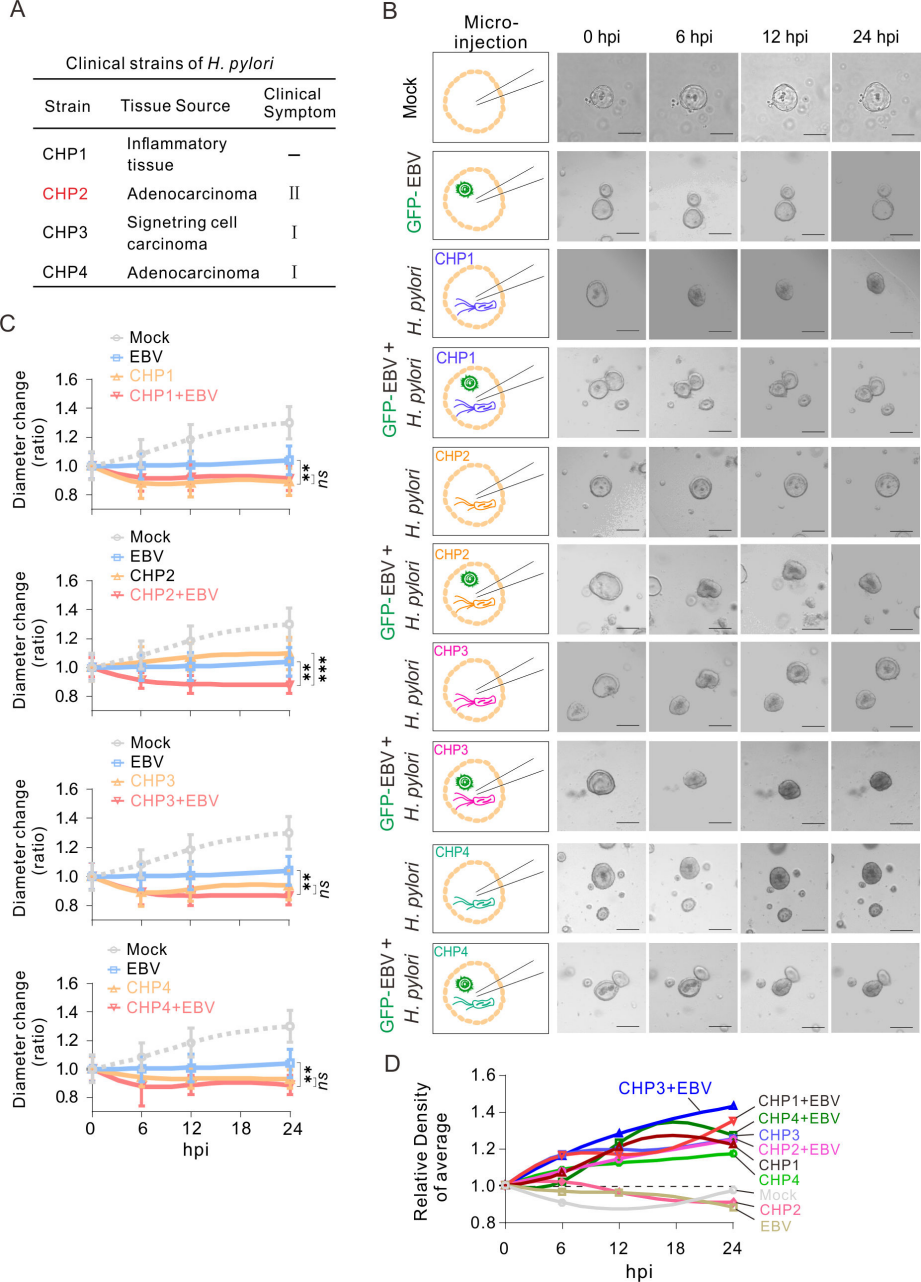
Effect of different *H. pylori* clinical strains co-infected with EBV via micro-injection on gastric organoid morphology and growth. (**A**) Resource of different clinical strains of *H. pylori*. (**B**) NGOs were individually micro-injected with GFP-tagged EBV virion particles (MOI = 10) and different clinical strains of *H. pylori* from panel A or both as indicated in the left panels, and morphology was visualized by microscopy analysis at 0, 6, 12, and 24 h post-infection (hpi). Scale bar, 100 µm. (**C**) The diameter ratio of NGOs micro-injected with *H. pylori* and GFP-EBV from panel B was evaluated by cross-section diameter analysis. (**D**) The relative density of NGOs micro-injected with GFP-EBV and *H. pylori* was evaluated based on the organoid morphology from panel B. The statistical analysis was done by ANOVA. **,* P *< 0.01, ***, *P *< 0.001, *ns*, not significant.

The *H. pylori* strain NCTC11637, which contains the complete virulence gene, exhibits a distinctive alteration of morphology and cell density in NGOs co-infected with EBV. We speculated that the effect of co-infection of EBV with distinct strains on NGOs could serve as a means to evaluate the virulence of the clinical strain *H. pylori*. Four strains (CHP1, CHP2, CHP3, and CHP4) of *H. pylori* from gastric tissues of patients, who exhibited disparate clinical symptoms and tumor types, were isolated and tested for co-infection (Fig. 3A). Interestingly, the results showed that each *H. pylori* strain (CHP1, CHP2, CHP3, and CHP4) infection alone or co-infection with EBV led to reduced growth of NGOs, when compared to the mock group (Fig. 3B and C). In contrast, co-infection of each *H. pylori* strain, including CHP1, CHP2, CHP3, and CHP4, with EBV consistently induced changes in morphology and higher organoid cell density along with prolongation of infection time within a 24 h period when compared to the infection of EBV or *H. pylori* alone control groups (Fig. 3D). Unexpectedly, the infection alone of CHP3 and CHP4, which is related to carcinoma, displayed a similar phenomenon, indicating that NGOs may represent a potential system to evaluate the toxicity of *H. pylori* strains with or without EBV co-infection in the clinic.

### EBV co-infection promotes the entry of *H. pylori* into NGOs

**Fig 4:**
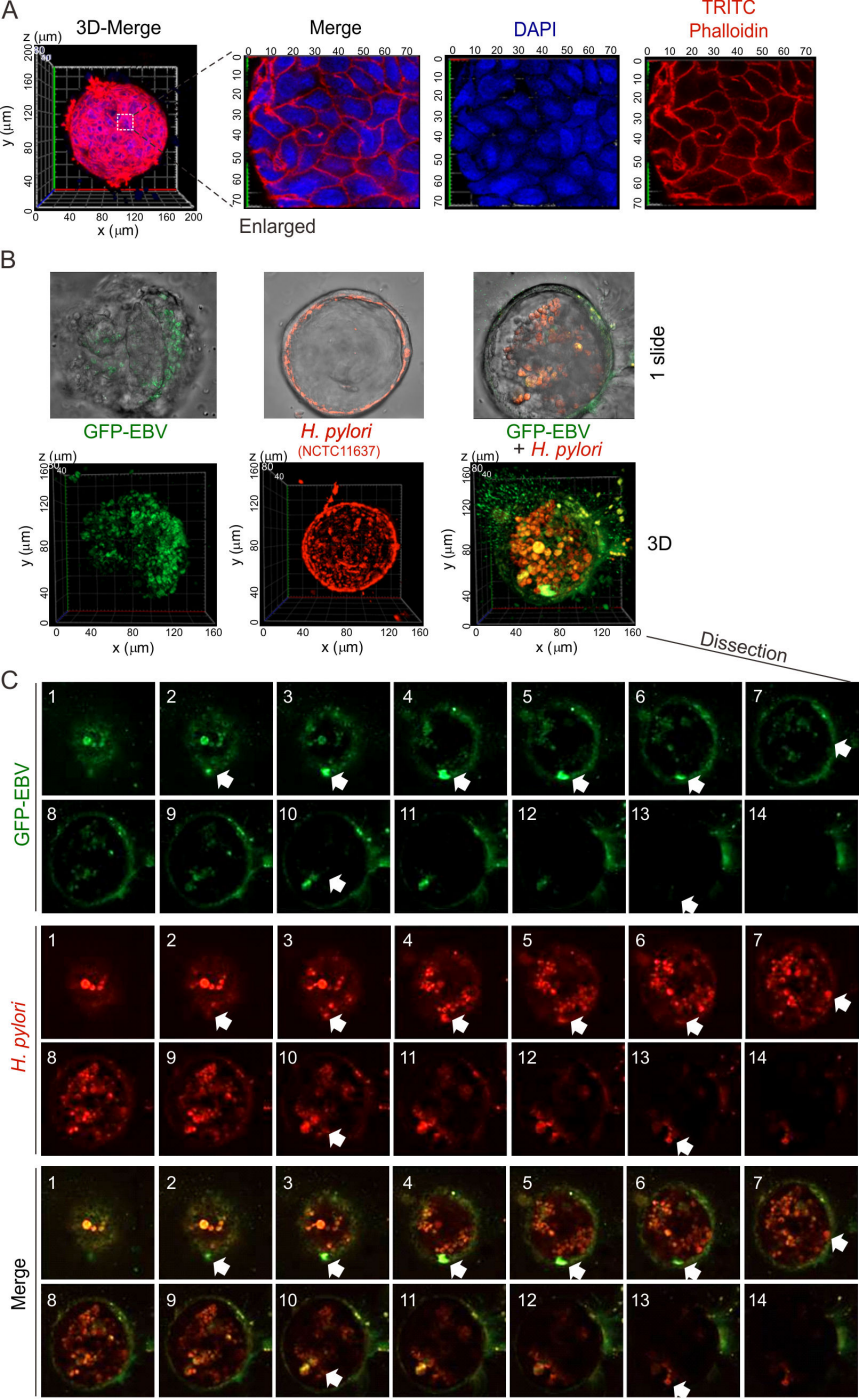
Co-infection of EBV promotes the internal localization of *H. pylori* from the gastric organoid surface. (**A**) Three-dimensional image of NGOs stained with TRITC phalloidin and DAPI for the nuclear compartment by immunofluorescent ZEISS Airyscan microscopy. (**B**) Co-infection of EBV promotes the internal localization of *H. pylori* in organoids. Bottom panel: three-dimensional images of NGOs micro-injected with GFP-tagged EBV and red CMTPX-dyed *H. pylori* strain NCTC11637 or both by immunofluorescent ZEISS Airyscan microscopy at 24 h post-microinjection. Scale bar, 40 µm. (**C**) Enlarged dissection images of 3D NGOs co-infected with GFP-EBV and *H. pylori* strain NCTC11637 in panel B. The co-location dots of *H. pylori* with EBV were highlighted by arrows.

**Fig 5:**
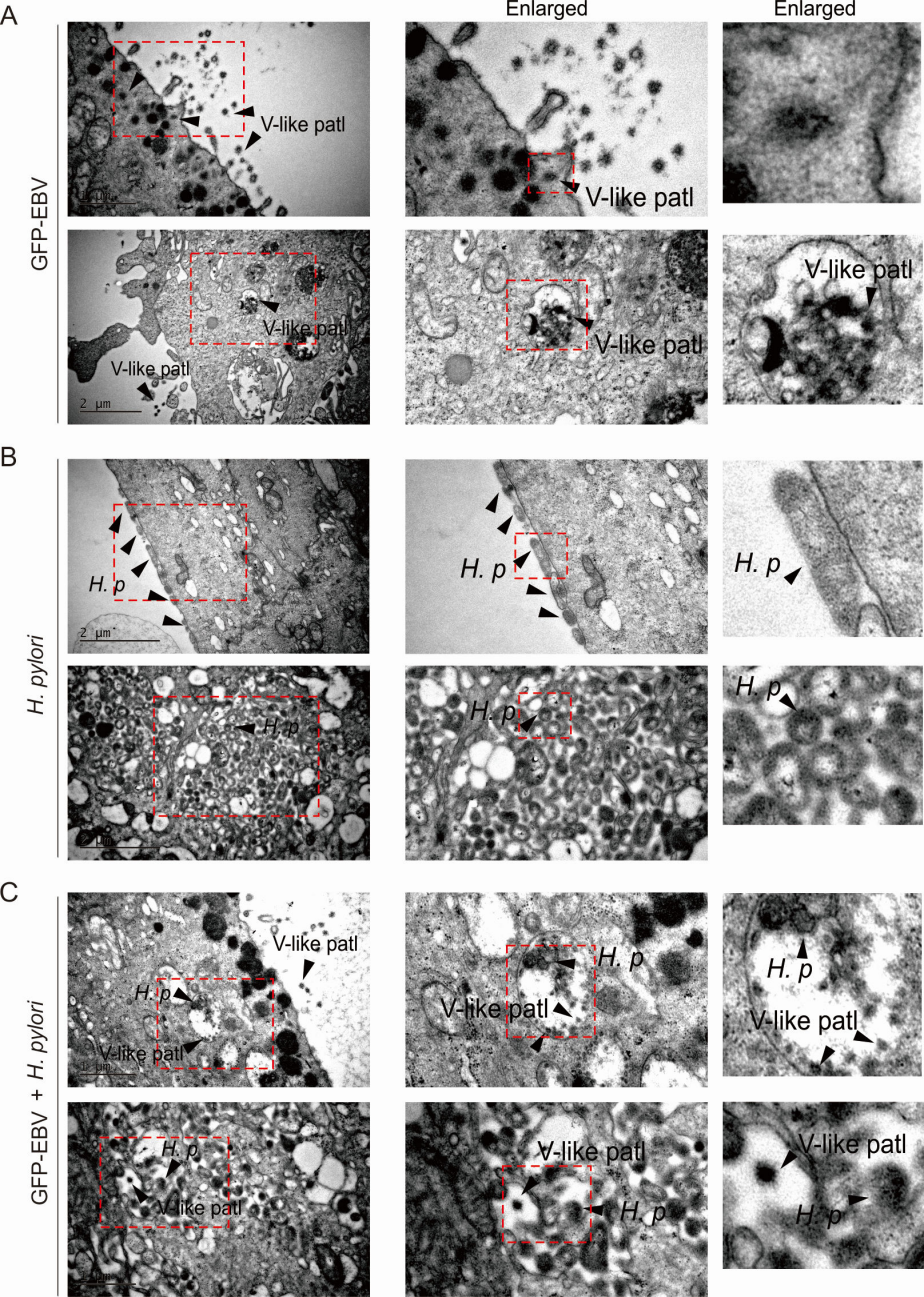
Transmission electron microscopy analysis of gastric organoids infected with EBV and *H. pylori*. NGOs were individually micro-injected with (**A**) GFP-tagged EBV virion particles (MOI = 10), (**B**) *H. pylori* strain (NCTC11637), or (**C**) both and subjected to transmission electron microscopy (TEM) analysis at 24 h post-infection (hpi). *V-like patl*: EBV-like particles; *H. p: H. pylori*. The locations of *H. pylori* and EBV-like particles are depicted by arrows.

To further address the role of EBV co-infection in the *H. pylori*-induced development of gastric cancer, we infected NGOs with the *H. pylori* strain NCTC11637 in the presence or absence of EBV, followed by three-dimensional stereoscopic imaging at 24 h post-infection. As shown in Fig. 4A, NGOs presented a complete three-dimensional spherical structure consisting of tightly interconnected gastric gland cells. Interestingly, when NGOs were infected with EBV alone, we observed that EBV infection within the lumen of the NGOs displayed a biased distribution, which tended to cluster in a particular region of the lumen of the NGOs and rarely on the other side (Fig. 4B, left panels). In contrast, when infected with the *H. pylori* strain NCTC11637 alone, *H. pylori* showed a non-biased infection on the surface of the organoid lumen, which tended to locate uniformly across the surface of the organoid lumen (Fig. 4B, middle panels). Interestingly, when the NGOs were infected with both EBV and *H. pylori*, although EBV still presented a biased infection status, the pattern of *H. pylori* location shifted from the majority of the surface to inside of the organoid and predominantly co-localized with the EBV-infected cells (Fig. 4C). To further confirm this phenomenon, we also performed transmission electron microscopy (TEM) analysis of NGOs with similar infections. Consistent with previous discoveries, the results revealed structures—morphologically reminiscent of EBV-like particles—and *H. pylori* cells apparently tethered to the apical (luminal) surface of the organoids; some of these structures seemed to breach the membrane, undergo apparent replication, and become enclosed in vesicles, suggestive of phagocytosis (Fig. 5A and B). Meanwhile, NGOs appeared to accumulate micro-villi and enzyme particles at the site where EBV-like structures colonized with *H. pylori* in the co-infection group, but did not appear in the *H. pylori* alone group (Fig. 5C). Collectively, these images tentatively suggest that EBV co-infection may augment *H. pylori* association with gastric epithelia, although definitive identification of the virus-like structures awaits further investigation.

### *H. pylori* co-infection enhances expressions of both EBNA1 and ZTA encoded by EBV

**Fig 6:**
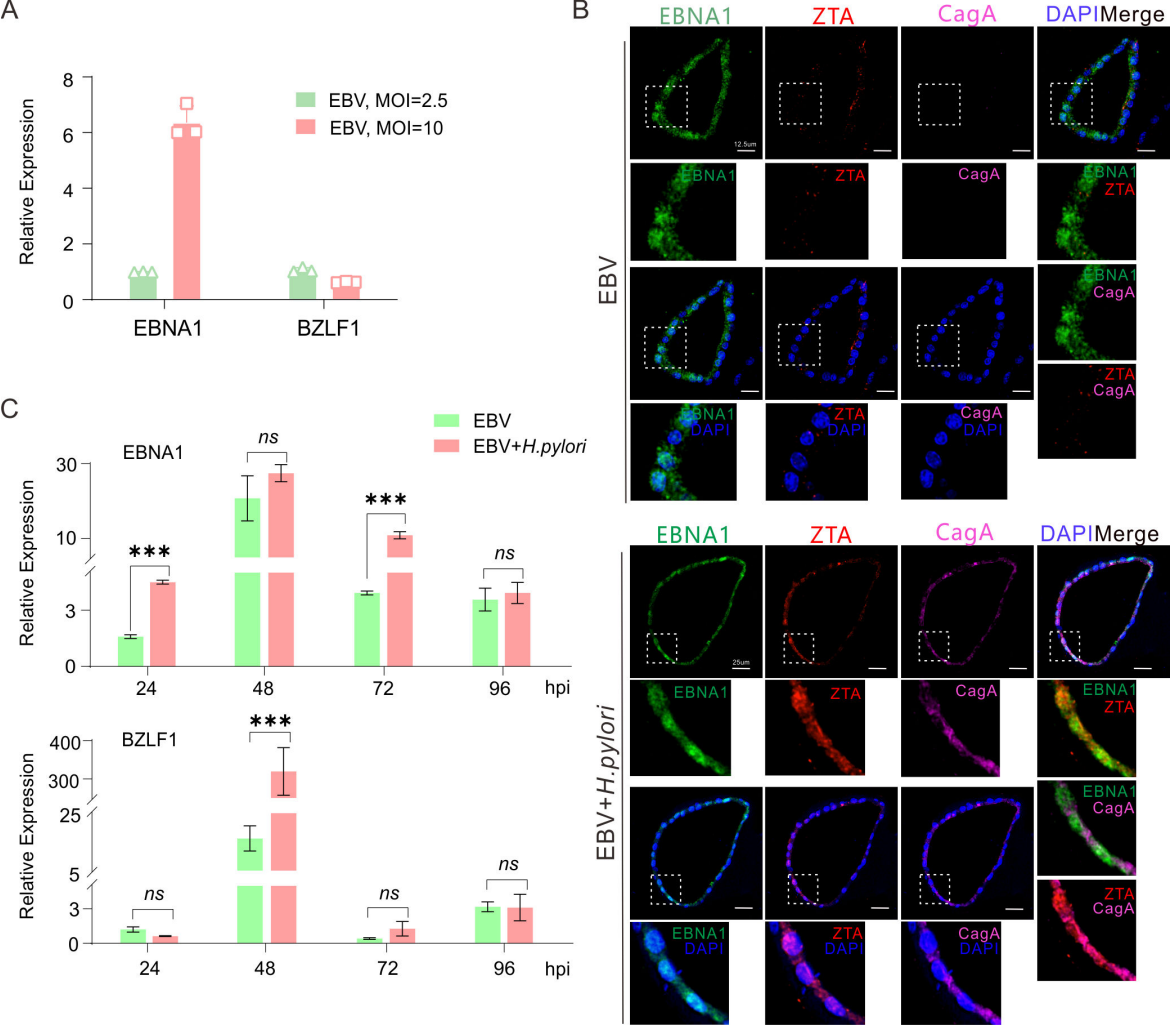
Expressions of EBNA1 and BZLF1 are enhanced in the EBV-infected gastric organoids with *H. pylori* co-infection. (**A**) Total RNA extracts from NGOs individually micro-injected with GFP-tagged EBV virion particles at low (MOI = 2.5) or high (MOI = 10) doses at 24 h post-infection were subjected to quantitative PCR analysis of EBNA1 and BZLF1. (**B**) Representative immunofluorescence images of NGOs immunostained with EBNA1, ZTA (BZLF1), and CagA. Cell nuclei were stained with DAPI. NGOs infected with EBV alone and co-infected with EBV and *H. pylori* are shown. MOI = 10. Scale bar, 12.5 µm (GFP-EBV only) and 25 µm (GFP-EBV co-infected with *H. pylori* strain NCTC11637). (**C**) Total RNA extracts from NGOs individually micro-injected with GFP-tagged EBV virion particles (MOI = 10) alone or co-infected with *H. pylori* strain (NCTC11637) at 24, 48, 72, and 96 h post-infection (hpi) were subjected to quantitative PCR analysis of EBNA1 and BZLF1. The statistical analysis was done by *t*-test. ***, *P *< 0.001*, ns,* not significant.

Since the infection of EBV in host cells has latent and lytic replication, to determine the status of EBV latent or lytic replication in NGOs co-infected with *H. pylori*, we first detected the expression levels of both EBNA1 (the key latent antigen) and BZLF1 (the master lytic activator) in NGOs infected with different dosages of EBV by quantitative PCR analysis. The results showed the high dosage (MOI = 10) of EBV infection could dramatically enhance the expression of EBNA1 when compared to low dosage (MOI = 2.5), while no significant difference in BZLF1 expression was found (Fig. 6A). Then, we carried out an immunofluorescence analysis of NGOs infected with EBV (at MOI = 10) alone or co-infected with *H. pylori*. Consistently, the results revealed that EBV alone predominantly expressed EBNA1 instead of the lytic activator ZTA (the protein encoded by BZLF1) when infecting NGOs (Fig. 6B, upper panels). In contrast, *H. pylori* co-infection in NGOs significantly enhanced the ZTA expression (Fig. 6B, lower panels), which was further supported by the results of the quantitative PCR analysis revealing that the expression level of EBNA1 in the co-infected group was consistently higher than that in the EBV alone group at different time points of infection (24, 48, 72, and 96 hpi) and peaked at 48 h post-infection, where the expression of BZLF1 was also significantly enhanced (Fig. 6C). These indicate that co-infection of *H. pylori* will enhance EBV latent infection and activate viral lytic replication during primary infection.

### Co-infection of *H. pylori* with EBV significantly impairs the expression of host genes related to cell proliferation and morphogenesis

**Fig 7:**
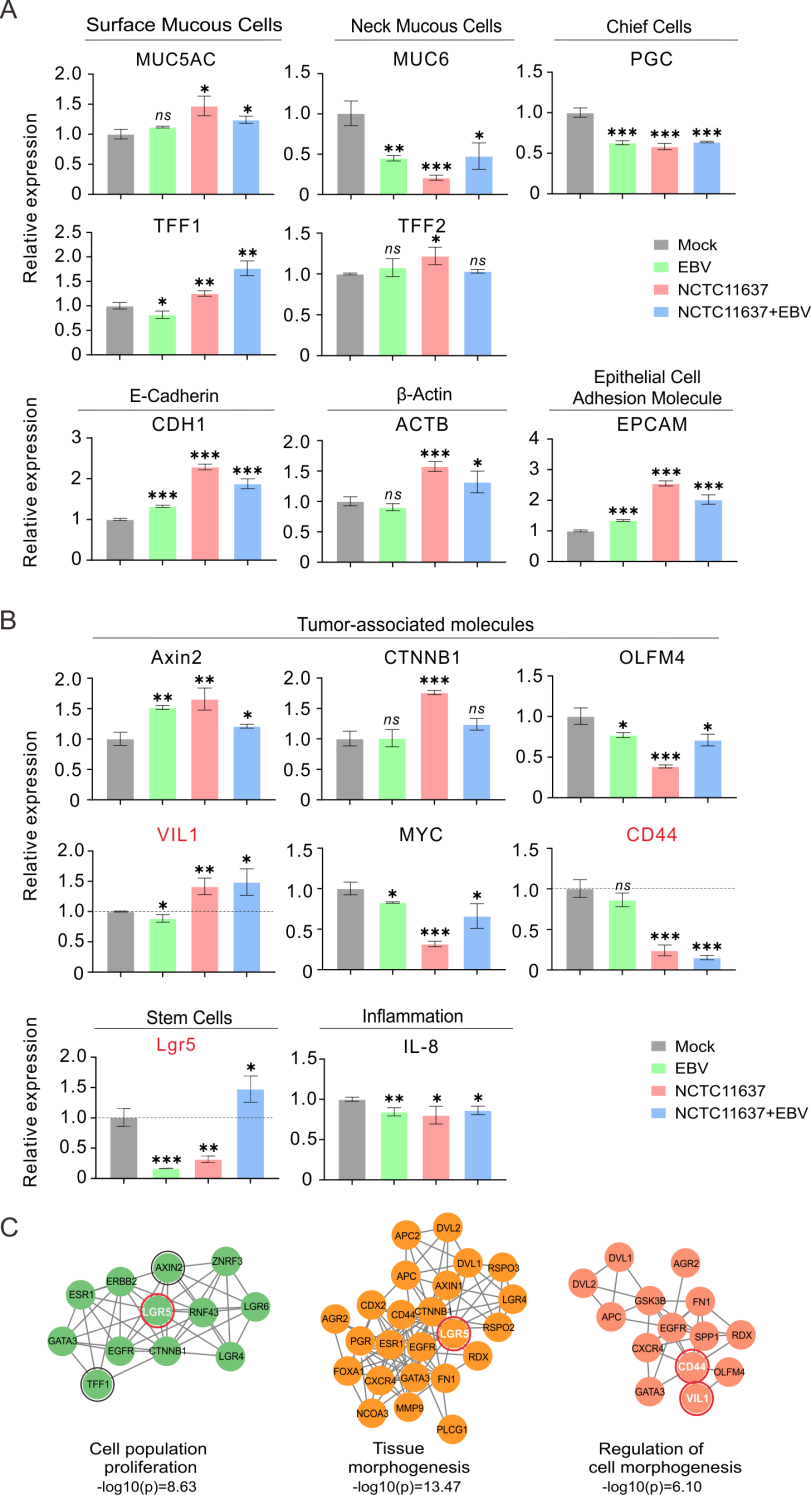
Expression profiles of the key biomarkers of gastric glands in gastric organoids infected with EBV and *H. pylori*. Total RNA extracts from NGOs individually micro-injected with GFP-tagged EBV virion particles (MOI = 10), *H. pylori* strain (NCTC11637), or both at 24 h post-infection were subjected to quantitative PCR analysis of biomarkers of (**A**) intrinsic gland components and (**B**) tumor-related molecules, stem cells, and inflammation. The statistical analysis was done by *t*-test. *, *P *< 0.05; **, *P* < 0.01; ***, *P < 0.001, ns, *not significant. (**C**) Functional enrichment of target genes with significant changes in expression in gastric organoids with co-infection of EBV and *H. pylori*.

Since *H. pylori* strain NCTC11637 co-infection with EBV impairs the growth of and morphology change in NGOs, we attempted to examine the expression of host genes related to glands and tumor biomarkers, including *MUC5AC* and *TFF1* (for surface mucous cells), *MUC6* and *TFF2* (for neck mucous cells), *PGC* (for chief cells), *CDH1*, *ACTB*, and *EPCAM* (for cytoskeleton), *Lgr5* (for stem cells), and *IL8* (for inflammatory), as well as *Axin2*, *CTNNB1*, *OLFM4*, *VIL1*, *MYC*, and *CD44* (for tumor genes). Consistently, the results showed that NGOs displayed a significant upregulation of *TFF1*, *VIL1*, and *Lgr5* at 24 h after co-infection of *H. pylori* with EBV and a downregulation of *CD44* and *Axin 2* compared to the *H. pylori* or EBV-alone groups (Fig. 7A and B). Functional cluster analysis further revealed that co-infection of *H. pylori* with EBV was involved in the regulation of cell proliferation and tissue morphogenesis (Fig. 7C), supporting our speculation that co-infection of EBV promotes *H. pylori*-mediated morphology and cell density changes in NGOs.

**Fig 8:**
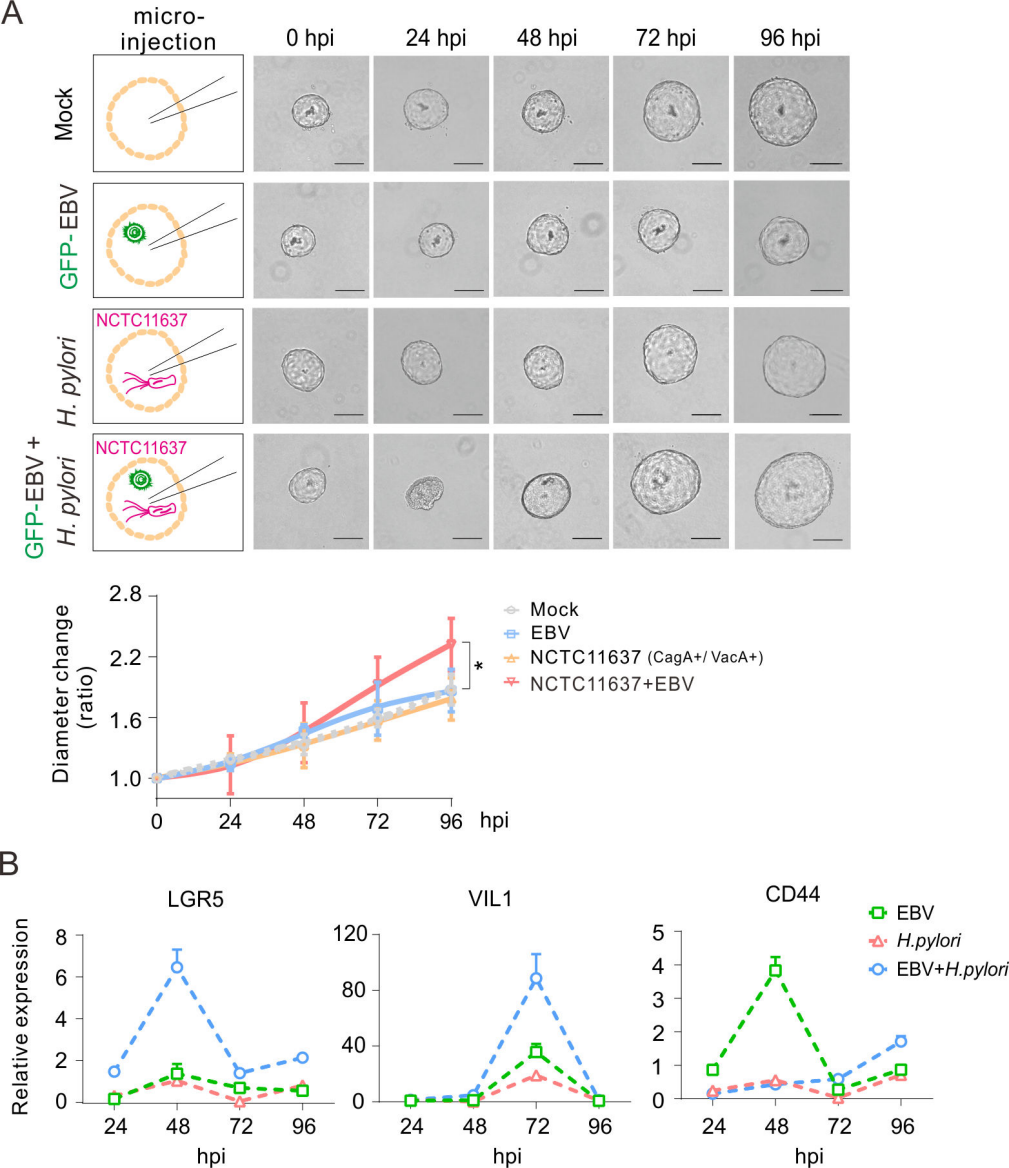
Real-time impact of *H. pylori*-EBV co-infection via microinjection on gastric organoid morphology, growth, and expression of key glandular biomarkers. (**A**) NGOs were individually micro-injected with GFP-tagged EBV virion particles (MOI = 10), *H. pylori* strain (NCTC11637), or both, and the organoid morphology is visualized by microscopy analysis at 0, 24, 48, 72, and 96 h post-infection (hpi). Scale bar, 100 µm. Bottom panel, the diameter ratio of NGOs micro-injected with *H. pylori*, GFP-EBV, or both was evaluated by cross-section diameter analysis. The statistical analysis was done by ANOVA. *,* P *< 0.05*.* (**B**) Total RNA extracts from NGOs individually micro-injected with GFP-EBV, *H. pylori*, or both from panel A were subjected to quantitative PCR analysis for the expression of key glandular biomarkers (Lgr5, VIL1, CD44).

To further confirm the role of EBV and *H. pylori* co-infection in NGOs, the morphology and growth status of NGOs and the expression patterns of key molecular markers, including Lgr5, VIL1, and CD44, were monitored every day for 4 days after single or co-infection. Compared to the EBV or *H. pylori*-only group, the results showed that NGOs underwent significant structural collapse within 24 h in the co-infected group; however, the three-dimensional organization of the cells gradually recovered the baseline morphology and continued to rapidly grow, which was supported by the evidence that the proliferation rate of NGOs exhibited a significant increase after 48 h post-infection and reached the most substantial growth at 96 h (Fig. 8A, lower panels). Using quantitative PCR analysis, the results showed that the expressions of Lgr5 and VIL1 gradually increased, peaking at 48 and 72 h in the co-infection of the EBV and *H. pylori* groups, respectively (Fig. 8B). In contrast, the expression pattern of CD44 exhibited no significant change within 96 h after co-infection when compared to the EBV-only group, supporting the notion that co-infection of *H. pylori* with EBV significantly impairs the expression of host genes related to cell proliferation and morphogenesis.

## DISCUSSION

**Fig 9:**
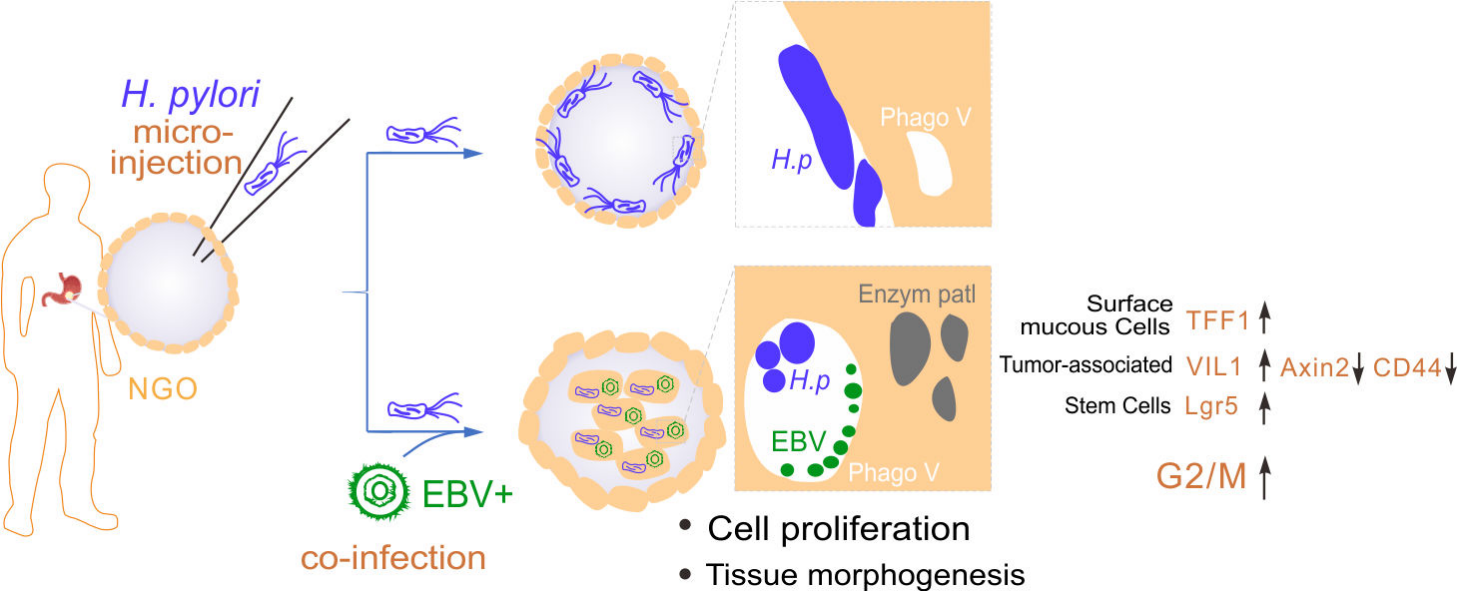
Schematic showing the role of *H. pylori* co-infected with EBV in cell proliferation and morphogenesis of gastric organoids. The co-infection of *H. pylori* with EBV promotes internal location of *H. pylori* and cell proliferation and morphogenesis of gastric organoids (NGO) via upregulating expressions of *VIL1* (tumor-associated molecule) and *Lgr5* (biomarker of stem cells) but downregulating the expression of *CD44* (tumor-associated molecule).

The gastrointestinal tract is a harsh environment that is typically inhospitable to most microorganisms. To adapt to this environment, microorganisms must tolerate extreme acidity, evade the action of digestive enzyme particles, and breach the self-protective barrier of the gastric mucosa to interact with the gastric epithelium and establish lifelong persistent infection. *H. pylori*, as a primary etiological agent, has been demonstrated to cause chronic inflammation and contribute to the development of GC, with a prevalence of >50% globally (33). The correlation between EBV and GC was initially reported in lymphoepithelioma-like carcinoma and gastric adenocarcinoma (34, 35), and increasing studies appear to suggest that *H. pylori* may play a synergistic role in the context of EBV-associated gastric cancer, which may involve potential mechanisms, including the following: (i) *H. pylori*-induced chronic inflammation and gastric mucosal lesions that create a microenvironment for EBV infection; and (ii) EBV and *H. pylori* co-infection promotes carcinogenesis by enhancing inflammatory responses and epigenetic alterations (20, 21, 36, 37). Nevertheless, the existence of synergistic mechanisms remains a contentious issue, as evidenced by the presence of site of action differences or independent/antagonistic effects (38). Moreover, the precise mechanism by which they co-infect the stomach remains to be elucidated due to the lack of suitable cell and animal infectious models *in vitro* and *in vivo*. In this study, for the first time, we infected patient-derived gastric organoids with EBV, *H. pylori*, or both using microinjection technology. Our results revealed that the co-infection of *H. pylori* with EBV not only dramatically enhances the expression of the EBV latent antigen EBNA1 and the key activator ZTA (encoded by BZLF1) for lytic replication, but also leads to a significant change in tissue structure (enhanced phagosome-like vesicle size, with co-localization of both *H. pylori* and EBV-like particles, and the accumulation of enzyme-like particles) and upregulates the expression of host genes, including *VIL1* and *Lgr5*, which are related to cell proliferation and tissue morphogenesis (Fig. 9).

Some groups have previously reported that *H. pylori* could play a role in facilitating EBV-induced proliferation of gastric epithelial cells by targeting epigenetic regulation of tumor suppressor genes (20) or by SHP1 phosphatase antagonizing the role of *H. pylori* CagA protein downregulation by EBV (36). We did observe a significant difference in the cell morphology of NGOs within 24 h post-infection in the *H. pylori* strain NCTC11637 instead of the SS1 group when they were co-infected with EBV, indicating that CagA is not a key factor involved in cooperating with EBV or innate immune response to induce cell morphogenesis in an organoid model. Nevertheless, the potential mechanisms underlying how co-infection with EBV and *H. pylori* accelerates tumor development and NGO morphology remain to be elucidated.

The advent of organoid technology has enabled the establishment of three-dimensional culture systems derived from a range of epithelial tissues, including the intestines (39, 40), esophagus (41), pancreas (42, 43), and liver (44–46). The gastric organoid model derived from the patient tissue exhibits a spherical structure that faithfully recapitulates the *in vivo* epithelial structure of the stomach’s monolayer columnar epithelium and its tissue-specific features; this allows the pathogen infection size to be manipulated in a specific location by micro-injection, as we have done in the central lumen compartment in this study. Furthermore, NGO can mimic the glandular structure and biological functions observed *in vivo* (47), making it an optimal choice for *in vitro* disease modeling in studying GC pathogenesis.

Although *H. pylori* is typically regarded as an extracellular bacterium, increasing evidence suggests that it is also capable of entering host cells, evading the host immune system attack, forming stable intracellular ecological niches, and being re-released into the extracellular milieu, which can result in recurrent infections (48–50). The initial objective of our study was to determine the localization of EBV and *H. pylori*, either as standalone infections or in a co-infected state, within the organoids. Unexpectedly, we found that EBV infection could result in altered localization of *H. pylori* within organoids and an increase in intracellular infection of *H. pylori*, which is consistent with the observations from the electron microscopy analysis showing structures morphologically suggestive of virus-like particles in close association with intracellular *H. pylori*. These findings further support the notion of bacterial internalization. Notably, we found that co-infection of EBV and *H. pylori* leads to an increased phagosome-like vesicle size and greater population of enzyme-like particles than that in the EBV infection alone group.

In addition, although it has been shown that *H. pylori* primarily relies on CagA for its virulence in the development of GC (10, 51, 52), and EBV can induce gastric mucosal lesions through the hypermethylation of CpG islands in the promoter regions of various cancer-associated genes (53), it is still largely unclear how the co-infection of *H. pylori* with EBV induces GCs. Our study showed for the first time that *H. pylori* co-infection with EBV markedly disrupted the globular structure of organoids, resulting in the curling and folding of organ tissues into irregular and disorganized shapes at the early stage (within 24 h post-infection) while increasing cell proliferation along with continued culture (up to 96 h post-infection). It is noteworthy to mention that the dramatically increased level of BZLF1 was observed in the co-infected group of *H. pylori* and EBV, which is distinct from the speculation that *H. pylori* may block EBV reactivation by inhibiting TGF-β1 from the clinic sample analysis (54). These findings indicate that co-infection with EBV and *H. pylori* has a deleterious effect on organ tissues. Moreover, the biomarkers of glandular intrinsic components (*TFF1*) and tumor-associated genes (*VIL1* and *Lgr5*) were significantly upregulated, providing further evidence that host aberrations and tumor progression are triggered by co-infection of EBV with *H. pylori*.

In summary, a three-dimensional co-infection system of EBV and *H. pylori* was constructed for the first time using gastric organ tissues derived from patients as an infection model. Similar to that, the organoid co-infection system has the potential to be employed for disease modeling, the evaluation of pathogen virulence, and the prediction of disease trends (11, 25, 55–57). Our findings not only offer new insights into basic and clinical translational research on the co-infection of different pathogens, but also have the potential to be employed as a tool to assess the virulence of *H. pylori* strains, developing treatment and predicting the progression of GC in clinics.

## MATERIALS AND METHODS

### Human subjects

Gastric tissues from gastric cancer patients (aged 55 to 86) were collected from Cancer Hospital of Fudan University. Redundant usage for research purposes was approved by the Hospital Medical Ethics Committee of Cancer Hospital of Fudan University. Sample size was based on the feasibility and availability of human excess tissue collections.

### Antibodies and reagents

Mouse antibody to Chromog A (60135-1-Ig, Proteintech), Amphiregulin (sc-74501, Santa Cruz), EBNA1 (sc-81581, Santa Cruz), ZEBRA (sc-53904, Santa Cruz), or CagA (sc-28368, Santa Cruz) was utilized. Rabbit antibody to TFF1 (13734-1-AP, Proteintech) or TFF2 (136811-1-AP, Proteintech) was used in this study. Alexa Fluor 488-conjugated goat anti-mouse IgG (H + L) (Cat#A11029) and Alexa Fluor 594-conjugated goat anti-rabbit IgG (H + L) (Cat #A-11012) antibodies were purchased from Invitrogen Co., Ltd. Multiple immunofluorescence with TSA fluorescent dye labeling was used for four-color development. The 4′,6-diamidino-2-phenylindole (DAPI) (Cat #C006, Solarbio), 2-O-tetradecanoylphorbol-13-acetate (TPA, Sigma), sodium butyrate (S615175, J&K), and TRITC Phalloidin (Yeasen) were used in this study.

### Bacterial strains

*Helicobacter pylori* strains SS1 (CagA+/VacA+), NCTC11637 (CagA+/VacA+), and NCTC12908 (CagA−/VacA+) were grown on columbia blood agar base (Oxoid) plates with 5% sheep blood (Jushi Biotech) in sealed anaerobic jars (MGC) with AnaeroPack-MicroAero bags (MGC) providing a micro-aerobic condition. To isolate a clinical strain of *H. pylori*, gastric surgery specimens were placed immediately in 250 µL 1× phosphate-buffered saline (PBS) (Meilunbio) at 4°C and homogenized using a tissue grinder. Fifty microliters was plated onto 5% sheep blood agar plate and incubated for 96 h under micro-aerobic conditions, as described. To count bacteria, *H. pylori* were scraped from blood agar plates and washed with PBS three times.

### Purification and quantitation of EBV virion

Akata EBV-positive cell lines carrying a GFP marker were used to generate virion particles of Epstein-Barr virus (EBV). Briefly, cells were sufficiently expanded in flasks and then cultured with 0.02 µg/mL TPA and 330 µg/mL sodium butyrate in 37°C and 5% CO_2_ in a humidified atmosphere for 3–4 days to lyse cells and release virus particles, followed by centrifugation at 2,000 ×*g* for 5 min and filtering through 0.45 µm membrane (Thermo). The supernatant was ultra-centrifuged at 29,000 ×*g* at 4°C for 2 h, and the virus pellet was re-suspended without serum or antibiotics, aliquoted, and stored at −80°C before use.

### *In vitro* cultivation of patient-derived gastric organoids

Human gastric organoids derived from patients were processed and obtained as described previously (32). Briefly, stomach surgery specimens were immersed and transferred in a balanced saline solution on ice. Micro-vessels, fatty tissue, and mesangium were removed under the microscope, and tissues were cut off into 2–5 mm^2^ and covered with 1× cold chelating buffer (5.6 mM Na_2_PHO_4_ [Sinopharm Chemical Reagent Co.]), 8.0 mM KH_2_PHO_4_ (Sinopharm Chemical Reagent Co.), 96.2 mM NaCl (Sinopharm Chemical Reagent Co.), 1.6 mM KCl (Sinopharm Chemical Reagent Co.), 43.4 mM sucrose (Sinopharm Chemical Reagent Co.), 54.9 mM D-sorbitol (Sangon Biotech), and 0.5 mM DL-dithiothreitol (AMRESCO) in sterile ddH_2_O, and then washed. The tissue pieces were pressed for three to five times until the supernatant became clear and incubated at room temperature after shaking (20–30 rpm) for 10 min supplemented with 2 mM EDTA in 1× chelating buffer. For gland isolation, the tissue pieces were gently pressed for three to five times using a sterile glass slide. The tissue debris was then suspended with basal medium (Advanced DMEM/F12 [Gibco]) added with 1% HEPES (Meilunbio), 1% penicillin/streptomycin, and 1% L-glutamine (Meilunbio), filtered through a 70 µm strainer (Thermo), and centrifuged at 400 ×*g* at 4°C for 5 min. For embedding and seeding, the gland debris was re-suspended with 1× Matrigel matrix (pheno-red free, Corning) and seeded in a pre-warmed plate. The plate was reversed and placed back in the incubator for Matrigel matrix three-dimensional structure solidifying. The organoids were cultured in growth medium of 50% WRN-conditioned medium supplemented with 50 ng/mL human recombinant EGF (Stemcell), 200 ng/mL human recombinant FGF (Stemcell), 1 nM Gastrin I (Stemcell), 2 µM A83-01 (Stemcell), 10 µM Y-27632 (Stemcell), 2% NeuroCult SM1 neuronal supplement (Stemcell), 1% N2 Supplement-A (Stemcell), and 1 mM N-acetyl-L-cysteine (Sigma). The medium was changed every 3 days. Transmitted light photographs were imaged with an inverted fluorescence microscope (EVOS).

### Immunofluorescence assays

The gastric organoids were fixed with 4% paraformaldehyde (PFA, Sigma) at room temperature for 1 h and immersed with 0.3% Triton X-100 (Beijing Lablead Biotech) at room temperature for 30 min or at 4°C overnight, followed by incubation with 0.05% fish skin gelatin (#G-7765, Sigma) at room temperature for 1 h or at 4°C overnight for blocking. The organoids were incubated with the indicated primary and secondary antibodies and nuclear staining with DAPI and then visualized with a laser scanning confocal microscope (TCS SP8, Leica).

### Quantitative PCR

Total RNA from gastric organoids was extracted and purified by using EZBioscience EZ-press RNA Purification Kit (EZB) and reverse-transcribed to cDNA using Hifair III First-Strand cDNA Synthesis SuperMix for qPCR Kit (Yeasen) according to the manufacturer’s instruction. The cDNA was amplified in a 20 µL total volume with 10 µL SYBR green, 0.4 µL each primer (10 µM), 4.2 µL H_2_O, and 5 µL cDNA by using Hieff qPCR SYBR Green Master Mix Kit (Yeasen) with specific primers. A melting curve analysis of the amplified products was carried out on a CFX Maestro System (Bio-Rad). Relative levels of gene expression were calculated using the threshold cycle (ΔΔCT) method with GAPDH as reference gene. The PCR amplification products were electrophoresed (120 V, 40 mA) in 3% agarose gel with nucleic acid dye for 40 min and visualized by the imager equipment (Tanon, Shanghai).

### Microinjection

Patient-derived gastric organoids were sub-cultured in homogenization before microinjection. Briefly, organoids were collected as normal sub-culturing procedures and centrifuged at 400 ×*g* at 4°C for 5 min, and then organoid pellets were incubated with 1× TrypLE Express (Gibco) in 37°C for 20 min to disassociate organoids into single cells (>80%). Cells were embedded with Matrigel matrix and cultured with 50% WRN-conditioned medium without antibiotics in the incubator for 2 days. The homogenized gastric organoids were microinjected with different pathogens by using a microinjection platform system (Eppendorf).

### Calculation of multiplicity of infection (MOI)

For calculation of the MOI, the organoids were disrupted into single cells by EDTA to count the cell number (approximately 4,000 cells per organoid). Bacterial counts were determined by measuring the optical density at 550 nm (OD_550_ = 0.1 equals to *H. pylori* concentration of 1 × 10^7^ CFU/mL). Viral particles were quantified by quantitative PCR targeting the EBNA1 gene, and the resulting concentrations were calculated according to the protocol described previously (58). The bacteria and virions were diluted to the appropriate concentrations and injected in the corresponding volumes (approximately 0.2 µL per organoid).

### Transmission electron microscopy

The gastric organoids were fixed, dehydrated, permeabilized, embedded, and imaged using a transmission electron microscopy system (Tecnai G2 Spirit TWIN; FEI). For the sample preparation, the organoids with the Matrigel matrix in the plate were pipetted gently for two to three times (not to break organoids), and the plate was put on ice for 10 min (for gel melting). The organoid suspension was then transferred to a conical tube (Thermo), centrifuged at 300 ×*g* (4°C, 5 min), and washed with cold 1× PBS for three times. Gastric organoids were fixed with 2.5% glutaraldehyde, post-fixed with 1% osmium tetroxide, subjected to graded dehydration with ethanol and acetone, cured in an oven, cut into ultrathin sections (50–60 nm) using LEICA UC7, stained with uranyl acetate and lead citrate, and examined in a PHILIPS CM-120 TEM.

### Cell cycle assay

The cell cycle assays of organoids were carried out as described previously (59). Briefly, the organoid cells were harvested, washed twice with PBS and resuspended, and fixed in 70% ethanol. The fixed cells were washed twice with PBS, and then 1 mL propidium iodide (PI) staining solution (50 µg/mL) and RNase A were added to the cell pellet for incubation for 20 min at room temperature. The 1.5 × 10^4^ PI-stained cells were subjected to flow cytometry by using BD FACS Calibur and analyzed by FlowJo software.

### Function enrichment analysis of target genes

The expression of target genes with significant change (including TFF1, VIL1, Lgr5, Axin2, and CD44) after co-infection of *H. pylori* and EBV was subjected to analysis using the STRING database (https://cn.string-db.org/). The input gene symbol was converted to the Entrez Gene ID of *Homo sapiens* using Metascape (http://metascape.org/) prior to subsequent bioinformatics analysis. The Gene Ontology (GO) of the biological process pathway was employed to extract annotation data from the gene list. Functional enrichment analysis was conducted to ascertain the prevalence of these genes within specific biological processes. The following criteria were established for the screening process: the minimum of three genes, the *P*-value cutoff of 0.01, and the minimum enrichment value of 1.5. The application of these criteria resulted in the identification of the top 20 significantly enriched biological pathways. The key functional pathways related to target genes were visualized using Cytoscape software (v3.10.1) to construct network diagrams illustrating the interactions between the genes.

### Statistical analyses

Statistical analyses were performed using the unpaired two-tailed Student’s *t* test (GraphPad Prism 9.0 and ImageJ software) or two-way analysis of variance (ANOVA) in the presence of a time factor. Each experiment was performed at least three times, and the mean and standard deviation were calculated. Differences were considered significant at *P *< 0.05*.*

## Data Availability

All data from this study are included within this article and are available from the lead contact (Qiliang Cai, qiliang@fudan.edu.cn) upon request.
